# Intracranial metastasis following intrahepatic remission in primary hepatic small cell neuroendocrine carcinoma: a case report

**DOI:** 10.3389/fmed.2026.1928042

**Published:** 2026-10-02

**Authors:** Jing Cui, Yukai Gao

**Affiliations:** Department of Oncology, Binzhou Central Hospital, Binzhou, Shandong, China

**Keywords:** case report, Hepatogenic small cell neuroendocrine carcinoma, Immune checkpoint inhibitor, Immunotherapy, multimodal therapy

## Abstract

**Background:**

Hepatogenic small cell neuroendocrine carcinoma (HP-SCNEC) is a highly aggressive rare malignancy, with central nervous system (CNS) metastasis being a major cause of treatment failure. This case report describes a patient with extensively metastatic HP-SCNEC, aiming to explore the clinical features of rapid brain metastasis following complete remission of intrahepatic lesions, as well as corresponding multidisciplinary treatment strategies.

**Method:**

A retrospective analysis of the diagnostic and therapeutic process in a 60-year-old female patient with HP-SCNEC. At initial presentation, the patient exhibited multiple intrahepatic metastases, abdominal lymph node involvement, and adrenal metastases. First-line therapy comprised the programmed death receptor-1 (PD-1) inhibitor sintilimab combined with etoposide and cisplatin. Following disease progression, whole-brain radiotherapy was administered for newly identified brain metastases, concurrently with systemic treatment adjustment to albumin-bound paclitaxel combined with anlotinib.

**Results:**

Following 6 cycles of immunotherapy combined with chemotherapy and 2 cycles of immunotherapy combined with anti-angiogenic therapy, the patient achieved radiographic complete remission of hepatic lesions. However, merely 16 days after achieving this remission, the patient presented with new-onset neurological symptoms, and subsequent imaging confirmed multiple new brain metastases. After undergoing whole-brain radiotherapy combined with an adjusted systemic treatment regimen, intracranial lesions were effectively controlled. The patient remained stable during subsequent maintenance therapy with Anlotinib.

**Conclusion:**

This case demonstrates that even when HP-SCNEC abdominal lesions achieve deep remission through intensified immunotherapy combined with chemotherapy, patients remain at extremely high risk for early brain metastases, highlighting the “intracranial sanctuary” effect caused by the blood-brain barrier. For such patients, prevention and monitoring of central nervous system metastases must be prioritized during initial treatment. Following brain metastasis, a multimodal comprehensive strategy centered on radiotherapy combined with intracranial active drugs should be adopted. This case provides important reference for the comprehensive management of this rare malignant tumor.

## Introduction

Hepatic Primary Small Cell Neuroendocrine Carcinoma (HP-SCNEC) is a rare malignant tumor originating in the liver with highly aggressive biological behavior. According to the World Health Organization Classification of Tumors of the Digestive System (5th Edition), this tumor is classified as high-grade neuroendocrine carcinoma (NEC, G3), characterized by extremely high cellular proliferation activity (Ki-67 index typically >50% in reported cases), rapid clinical progression, and extremely poor prognosis (1). Due to its extremely low incidence—accounting for only 0.3%–0.5% of all liver malignancies—currently no evidence-based high-level clinical guidelines exist. Management strategies are largely derived from research data on small cell lung cancer (SCLC) and high-grade NEC at other sites, which exhibit similar biological behavior (2, 3).

The diagnosis of HP-SCNEC relies on histopathological examination and immunohistochemical staining. Typical morphological features include small round or oval tumor cells with fine chromatin and active mitotic activity. Immunohistochemistry typically demonstrates positive expression of neuroendocrine markers such as synaptophysin (Syn), chromogranin A (CgA), and CD56, while epithelial markers like cytokeratin (CK) are also frequently positive (4). The patient's pathology in this case meets the aforementioned criteria (Syn+, CD56+). It is noteworthy that since metastatic neuroendocrine tumors to the liver are far more common than primary liver tumors, diagnosing a primary liver tumor necessitates first ruling out primary lesions in other sites—particularly the lungs, pancreas, and gastrointestinal tract—which adds to the challenge of clinical diagnosis (5).

For resectable localized HP-SCNEC, radical hepatectomy represents a potentially curative approach. However, clinically, over half of patients present with multiple intrahepatic or distant metastases at initial diagnosis, thereby losing surgical eligibility (6, 7). For advanced-stage patients, systemic systemic therapy constitutes the primary treatment strategy. Epirubicin combined with platinum-based chemotherapy (EP/EC regimen) is a traditional first-line treatment derived from SCLC therapy. In advanced high-grade NEC, it achieves an objective response rate (ORR) of 30%–70%, but the median progression-free survival (PFS) is typically short (approximately 4–6 months), and the vast majority of patients ultimately experience disease progression (8, 9).

With the advancement of tumor immunotherapy, immune checkpoint inhibitors such as programmed death receptor-1/ligand-1 (PD-1/PD-L1) inhibitors have brought new hope for treating various solid tumors, including certain neuroendocrine tumors. Although neuroendocrine tumors are typically regarded as “immunologically cold tumors” characterized by low tumor mutational burden and minimal tumor-infiltrating lymphocytes (TILs), recent studies have revealed that high-grade neuroendocrine carcinoma (G3) may exhibit greater immunogenicity and higher PD-L1 expression levels than low-grade neuroendocrine tumors (NETs). This finding provides a theoretical basis for the application of immunotherapy in this subgroup (10, 11). Multiple clinical studies have begun investigating the efficacy of immunotherapy as monotherapy or in combination with chemotherapy for advanced NEC. Results indicate that it can induce durable clinical responses in some patients, but the overall response rate still needs improvement (12, 13).

Anti-angiogenic therapy represents another crucial systemic treatment strategy. Tumor growth and metastasis depend on the formation of new blood vessels. Multi-targeted tyrosine kinase inhibitors (TKIs), exemplified by anlotinib, effectively inhibit targets such as vascular endothelial growth factor receptors (VEGFR). These agents have demonstrated favorable disease control rates and survival benefits in multiple clinical trials for advanced neuroendocrine tumors (14, 15). More importantly, basic and preclinical studies indicate that anti-angiogenic drugs can exert synergistic effects with immune checkpoint inhibitors by modulating the tumor immune microenvironment—such as increasing effector T cell infiltration and reducing immunosuppressive cells—a phenomenon termed the “immunoregulatory normalization” effect. This provides a robust scientific rationale for their combined application (16, 17).

Central nervous system (CNS) metastasis is one of the primary causes of treatment failure and mortality in various aggressive tumors, including small cell lung cancer (SCLC) and high-grade neuroendocrine carcinoma (NEC). The blood-brain barrier (BBB) presents a significant physiological barrier to drug delivery, making it difficult for many macromolecular drugs (such as antibody-drug conjugates) and certain chemotherapeutic agents to achieve effective therapeutic concentrations in cerebrospinal fluid. Consequently, the CNS often serves as a “sanctuary” for tumor cells (18). When brain metastases occur, local therapies (such as whole-brain radiotherapy and stereotactic radiosurgery) form the cornerstone of treatment. However, to achieve more durable systemic and intracranial disease control, integrated management combining systemic therapies capable of partially crossing the BBB (such as certain chemotherapeutic agents and small-molecule TKIs) has become a key contemporary clinical strategy (19, 20).

Therefore, the treatment of advanced HP-SCNEC remains a complex and challenging field. The current focus of clinical research lies in integrating the advantages of traditional chemotherapy, immunotherapy, and anti-angiogenic therapy to develop optimal combination or sequential treatment regimens, aiming to maximize patients’ progression-free survival and overall survival. In particular, effectively preventing and treating brain metastases represents a critical bottleneck in improving long-term patient outcomes. This case report describes a patient with newly diagnosed, extensively metastatic HP-SCNEC who achieved complete hepatic response to first-line PD-1 inhibitor combined with EP chemotherapy. However, intracranial progression occurred within a short period. Subsequently, disease control was successfully achieved through whole-brain radiotherapy combined with chemotherapy and anti-angiogenic therapy. This process highlights the complexity of treating this disease, the limitations of existing regimens, and the importance of multimodal integrated therapy. It aims to provide reference for clinical practice in this rare disease and offer insights for further optimizing treatment strategies.

### Patient information

Female patient, 60 years old at initial presentation, presented to our center on March 8, 2025, complaining of “upper abdominal distension and discomfort for 2 months”. The patient reported: Upper abdominal distension and discomfort appeared without obvious precipitating factors. Abdominal distension and discomfort worsened after meals. Occasional dull abdominal pain and discomfort were present, accompanied by a sensation of pulling discomfort in the lumbar and back regions.

### Clinical findings

On March 21, 2025, an ultrasound-guided liver biopsy was performed. Pathology findings: (Liver tumor biopsy specimen) Poorly differentiated carcinoma. Based on immunohistochemistry, consistent with neuroendocrine carcinoma (small cell carcinoma).

### Diagnostic assessment

Immunohistochemistry: Syn(+), CD56(+), GPC-3 (focal+), Arg-1 (-), HSP70 (sporadic+), CK7(+), CK19 (weak+). 2025-3-18 CT findings: Multiple intrahepatic lesions; enlarged lymph nodes in the abdominal cavity and retroperitoneum; right adrenal nodule with high likelihood of metastasis. Based on comprehensive examination results, the diagnosis is primary small cell neuroendocrine carcinoma of the liver with extensive metastasis (Figure 1). As part of the comprehensive staging workup, a contrast-enhanced brain MRI was also performed on March 18, 2025, which demonstrated no evidence of intracranial metastases, leptomeningeal enhancement, or any other abnormalities, confirming the absence of baseline central nervous system involvement.

**Figure 1:**
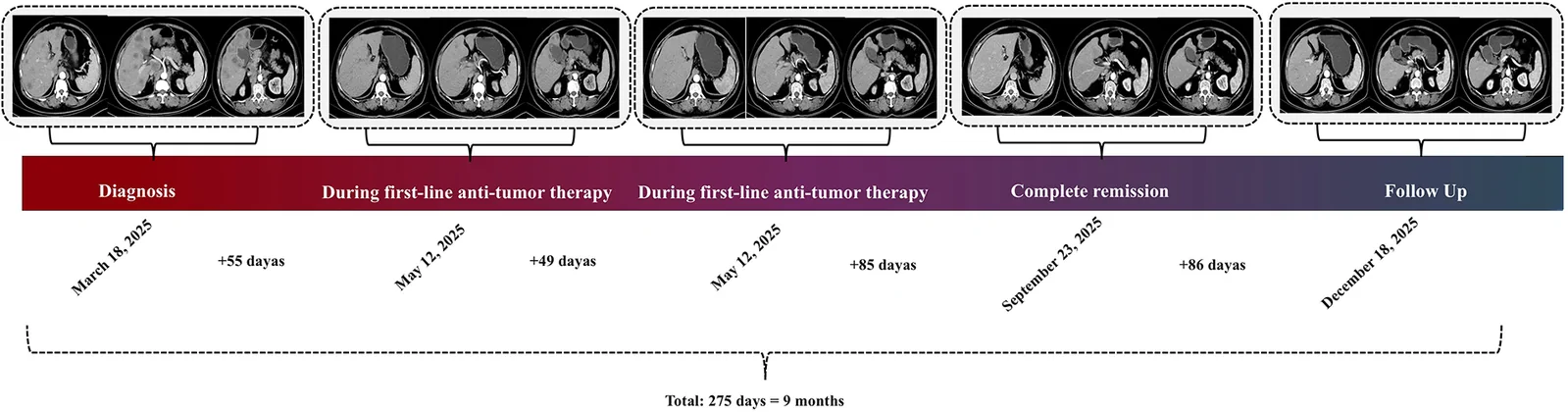
Timeline of changes in the primary liver cancer lesion.

### Therapeutic intervention

On March 19, 2025, the patient commenced a 6-cycle antitumor treatment regimen combining immunotherapy with chemotherapy, specifically: Sintilimab 200 mg ivgtt d1 + Etoposide 100 mg ivgtt d1-4 + Cisplatin 40 mg ivgtt d1-3. 2025-05-12 and 2025-06-30 CT scans both indicated disease improvement with tumor shrinkage (Figure 1). Subsequently, starting on August 21, 2025, the patient received two cycles of anti-angiogenic therapy combined with immunotherapy, specifically: Anlotinib 10 mg orally d1-14 q21d + Sintilimab 200 mg ivgtt. On September 23, 2025, CT imaging demonstrated the complete disappearance of the intrahepatic tumor, confirming radiographic complete remission (Figure 1). However, this remission proved to be extremely short-lived. Merely 16 days later, on October 9, 2025, the patient returned to our center with complaints of dizziness, fatigue, and malaise that had persisted for one week. An urgent MRI examination performed on October 10, 2025, unfortunately revealed multiple new metastatic lesions within the brain (Figure 2), indicating intracranial progression despite profound systemic disease control. Consequently, on October 13, 2025, whole-brain radiotherapy was initiated with a total dose of 40 Gy/20 fractions (2 Gy/fraction), concurrently administered with one cycle of chemotherapy combined with anti-angiogenic therapy. The specific regimen was: albumin-bound paclitaxel 300 mg ivgtt + anlotinib 10 mg. MRI examination following radiotherapy revealed multiple metastatic lesions in the brain. Compared with the MRI scan dated October 10, 2025, most lesions within the brain have decreased in size (Figure 2). Imaging revealed disease remission compared to previous assessments. Subsequently, the patient underwent two cycles of chemotherapy combined with anti-angiogenic therapy, specifically: albumin-bound paclitaxel 300 mg ivgtt + anlotinib 10 mg. Oral anlotinib therapy was continued until the last follow-up on December 18, 2025, with imaging showing stable disease (Figure 1).

**Figure 2:**
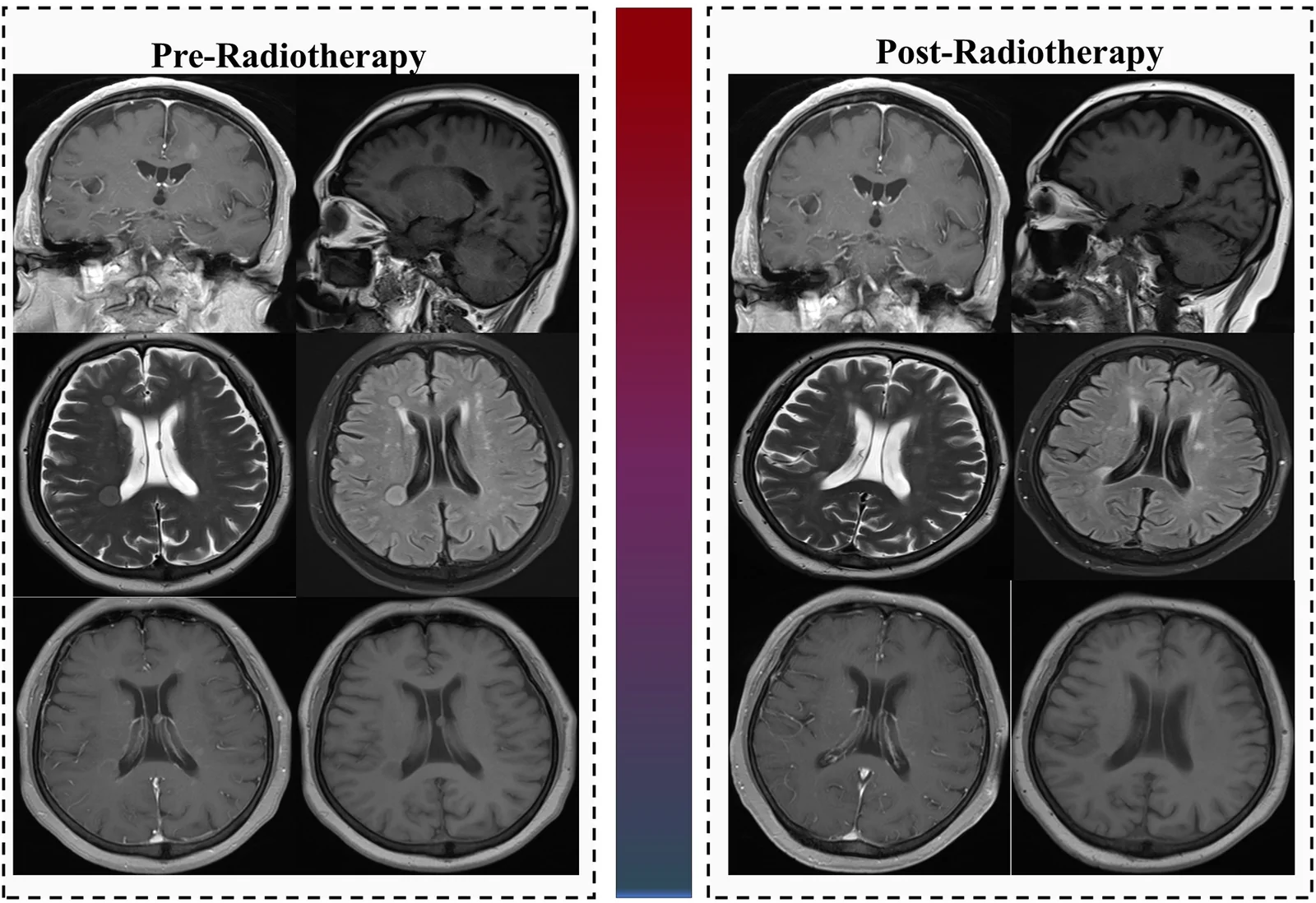
Changes in intracranial metastatic lesions before and after treatment.

## Discussion

This case report describes a patient with HP-SCNEC presenting at initial diagnosis with multiple intrahepatic lesions and extensive lymph node and adrenal metastases. The rarity of this case is evident on multiple levels: First, NENs themselves account for only 0.3%-0.5% of all liver malignancies; Second, the vast majority are well-differentiated NETs, whereas the high-grade neuroendocrine carcinoma (NEC, G3) in this case is exceptionally rare. Finally, among high-grade NECs, the small cell subtype—the most aggressive histological type—has only been sporadically reported in the global literature for primary liver cases, lacking systematic clinical data (1, 7, 21). Therefore, the diagnostic and therapeutic process in this case represents not only a response to a complex clinical challenge but also a valuable exploration of treatment strategies for an extremely rare, highly malignant solid tumor. The entire treatment journey can be clearly divided into two distinct phases, vividly reflecting the core approach of individualized, dynamic treatment decision-making based on tumor biology and multidisciplinary collaboration—particularly in the absence of established standard protocols.

A noteworthy diagnostic point deserves further discussion. The immunohistochemical panel in this case included hepatocyte-associated markers—GPC-3 (focal+), Arg-1 (-), and HSP70 (sporadic+)—in addition to the neuroendocrine markers. These markers were initially requested by the pathologist to rule out hepatocellular carcinoma (HCC), which was a consideration given the hepatic location of the tumor. The complete negativity of Arg-1 argues strongly against a hepatocellular origin. The focal GPC-3 positivity and sporadic HSP70 expression are best interpreted as either background staining from entrapped non-neoplastic hepatocytes adjacent to the tumor or, less likely, aberrant antigen expression in a subset of tumor cells—a phenomenon that has been occasionally described in non-hepatocellular malignancies but does not confer hepatocellular differentiation. Critically, the unequivocal and diffuse positivity for neuroendocrine markers (Syn, CD56), the characteristic small cell morphology, and the absence of any histological features of HCC (such as trabecular or pseudoglandular patterns, bile production, or cytoplasmic hyaline bodies) collectively support the diagnosis of pure small cell neuroendocrine carcinoma rather than a mixed hepatocellular-neuroendocrine tumor. Nevertheless, we acknowledge that the presence of these markers may cause diagnostic confusion, and we have provided this detailed pathological reasoning to guide interpretation.

At initial presentation, the patient was already in advanced metastatic disease with no surgical options available. The fundamental challenge for first-line therapy was selecting the systemic treatment regimen most likely to achieve rapid and deep disease control in the absence of a dedicated protocol for HP-SCNEC. The treatment team ultimately adopted an immunotherapy combination chemotherapy regimen of sintilimab + etoposide + cisplatin.

From the perspective of disease essence, HP-SCNEC exhibits high histological and biological similarity to SCLC, which is highly sensitive to EP chemotherapy regimens (8, 9). Therefore, the EP regimen constitutes a reasonable cornerstone for empirical treatment of this rare tumor. However, conventional chemotherapy demonstrates limited long-term control capacity for extensive-stage SCLC or high-grade NEC, with median PFS typically falling below 6 months (22). To overcome this bottleneck, combined immune checkpoint inhibitors have emerged as a promising strategy. Although neuroendocrine tumors are generally classified as “immunologically cold tumors,” evidence suggests that high-grade neuroendocrine carcinoma (G3) may exhibit a relatively higher tumor mutational burden and a more active tumor immune microenvironment, providing a potential basis for the efficacy of immunotherapy (11, 23). Recent early-phase clinical studies have also suggested that PD-1 inhibitors such as pembrolizumab demonstrate antitumor activity in some previously treated patients with metastatic high-grade NEC (12). Therefore, concurrent combination with a PD-1 inhibitor (sintilimab) in first-line therapy was hypothesized to leverage chemotherapy for rapid tumor cell killing and antigen release, while potentially utilizing immunotherapy to release T-cell suppression and establish durable immune memory. Theoretically, this approach may produce synergistic effects, although this remains a hypothesis rather than an established benefit in this disease context (24). Whether this strategy actually achieves deeper or more durable responses than chemotherapy alone in HP-SCNEC is unknown and requires formal investigation.

The treatment outcome of this case validates the efficacy of this exploratory regimen. The patient completed six cycles of therapy and achieved CR of the intrahepatic lesions in subsequent evaluations. However, the subsequent clinical course was particularly striking. The fact that intracranial metastases became clinically apparent a mere 16 days after achieving a complete radiographic response in the liver underscores the exceptionally aggressive biological nature of HP-SCNEC. This ultrashort interval provides compelling real-world evidence that while intensive first-line immunochemotherapy can effectively eliminate radiographically visible systemic lesions, it is largely incapable of eradicating micrometastatic deposits that have already seeded the central nervous system (CNS), likely due to the formidable pharmacological barrier imposed by the blood-brain barrier (BBB). This observation reinforces the concept of the CNS as a “sanctuary site” and highlights the critical need for early, proactive intracranial imaging surveillance, even in patients who demonstrate an otherwise excellent systemic response to therapy. This provides important clinical evidence and support for future prospective studies investigating immunotherapy combined with chemotherapy in other similar rare aggressive forms of NEC. However, this success has also raised new questions: For patients with extensive metastases who have achieved CR of abdominal lesions, how should the optimal duration of treatment and subsequent maintenance strategies (such as single-agent immunotherapy maintenance) be determined? This remains an unanswered question in current clinical practice.

Despite significant intraperitoneal success with first-line therapy, the patient developed multiple intracranial metastases within a short period. This pivotal shift vividly illustrates the classic therapeutic dilemma in treating highly malignant solid tumors like HP-SCNEC: the CNS's role as an “immunologically privileged” and “pharmacologically sheltered” environment (18, 25). The blood-brain barrier severely limits the effective concentration of most macromolecular drugs (such as monoclonal antibodies) and certain chemotherapeutic agents in cerebrospinal fluid. Consequently, even when systemic lesions are controlled, pre-existing circulating tumor cells or micro-metastases undetected by imaging may still “escape” intracranially and proliferate (26) (Figure 3).

**Figure 3:**
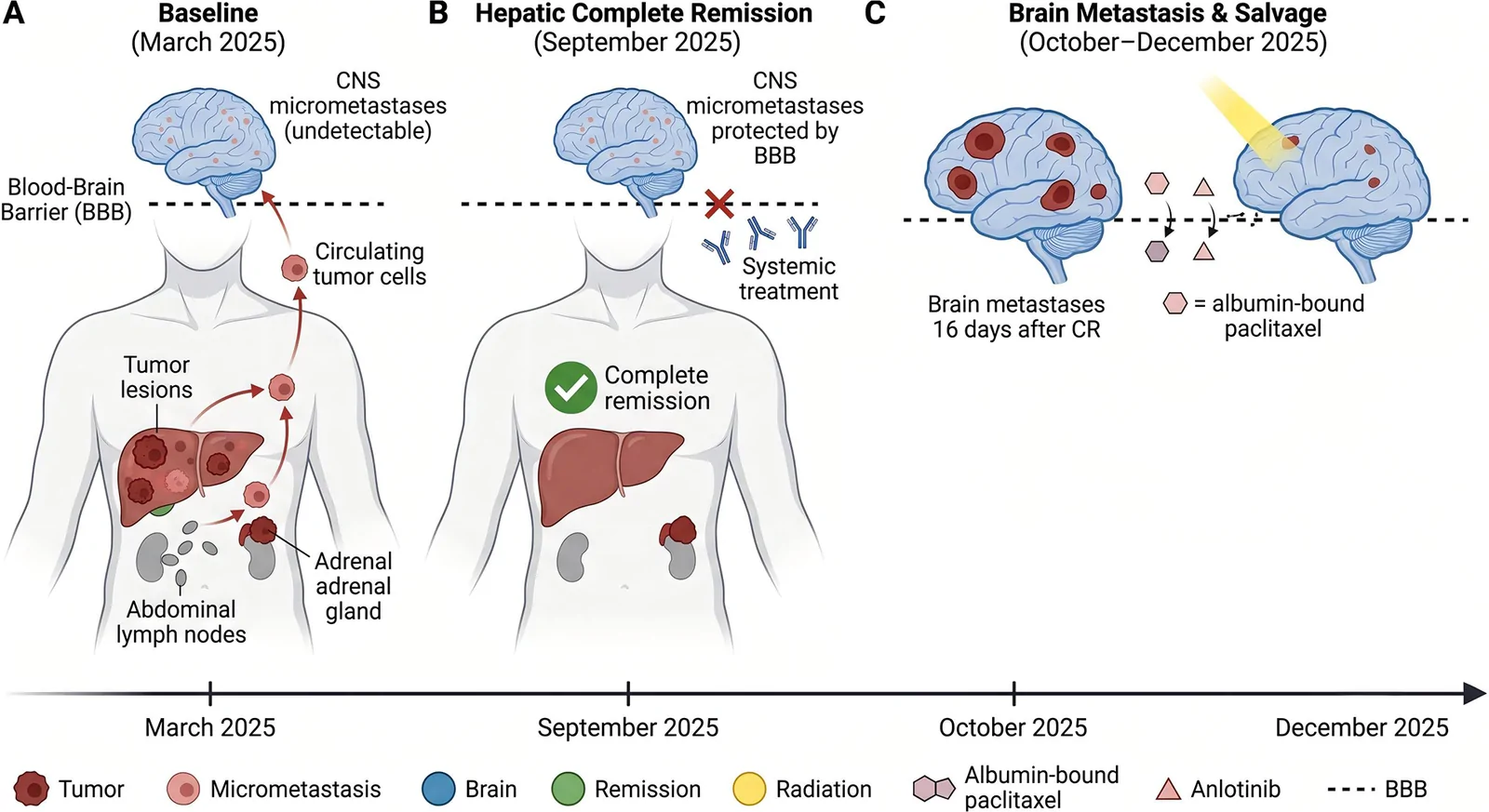
Hypothetical mechanistic model of the “intracranial sanctuary” effect in HP-SCNEC. **(A)** Baseline: extensive hepatic, nodal, and adrenal disease with hypothesized CNS micrometastatic seeding. **(B)** Hepatic complete remission after immunochemotherapy, with persistent CNS micrometastases protected by the blood-brain barrier (BBB). **(C)** Isolated brain metastases 16 days after hepatic complete remission; multimodal salvage with whole-brain radiotherapy plus albumin-bound paclitaxel and anlotinib.

When faced with intracranial progression, treatment strategies must immediately shift to multimodal comprehensive therapy targeting the CNS. The second-line regimen in this case exemplifies this principle:

Local curative treatment: For multiple brain metastases, whole-brain radiation therapy (WBRT, 40 Gy/20 fractions) serves as the standard local treatment modality. It aims to rapidly control visible lesions, prevent neurological dysfunction, and potentially create conditions for subsequent systemic drug penetration by disrupting the local blood-brain barrier (19).

Adjustments to Systemic Therapy: The team implemented targeted modifications to the systemic treatment regimen. Sintilimab and cisplatin, which struggle to cross the BBB, were discontinued and replaced with albumin-bound paclitaxel. Taxanes exhibit moderate lipophilicity, granting them superior BBB penetration compared to etoposide. Particularly during the window of increased BBB permeability following radiotherapy, taxanes may more effectively reach brain tissue to exert cytotoxic effects (27). Meanwhile, the role of anlotinib continues to be emphasized. As a small-molecule, multi-targeted TKI, anlotinib possesses a low molecular weight, theoretically enabling it to cross the BBB more readily than large-molecule antibodies. A growing body of evidence supports the value of such TKIs in controlling brain metastases from solid tumors such as NSCLC (28). In the field of neuroendocrine tumors, anlotinib has been demonstrated to exert dual effects of anti-angiogenesis and direct inhibition of tumor proliferation (15). Furthermore, anti-angiogenic agents may potentially produce long-term synergistic effects with prior immunotherapy by “normalizing” tumor vasculature and improving the immune microenvironment (16, 17).

Synergistic Treatment Modalities: This regimen represents not merely a simple combination of drugs, but embodies the synergistic concept of “local radiotherapy controlling overt lesions” and “modified systemic therapy eliminating micrometastases and preventing new ones”. Post-radiotherapy imaging revealed reduction in most brain lesions, with subsequent disease stability maintained under anlotinib maintenance therapy. This demonstrates the feasibility and efficacy of this multimodal strategy in controlling intracranial metastases of the rare tumor HP-SCNEC.

We acknowledge several important limitations in this case report. First and foremost, while the histopathological and immunohistochemical profile (Syn+, CD56+, CK7+, GPC-3 focal+) strongly supported the diagnosis of primary hepatic small cell neuroendocrine carcinoma, a chest CT, 18F-FDG PET-CT, or bronchoscopy was not performed to definitively exclude the possibility of an occult primary neuroendocrine tumor at more common sites, particularly the lung, pancreas, or gastrointestinal tract. Given that metastatic neuroendocrine tumors to the liver far outnumber primary hepatic neuroendocrine neoplasms, the diagnosis of HP-SCNEC in this case should be regarded as a “presumptive” rather than “definitively proven” primary hepatic origin. This limitation must be considered when interpreting the clinical course and treatment responses described herein. Second, this is a single case report with a relatively short follow-up duration, which limits the generalizability of our observations. Despite these limitations, we believe that the detailed characterization of this patient's aggressive clinical trajectory, rapid intracranial progression following systemic remission, and response to multimodal salvage therapy provides valuable insights for the clinical management of this ultra-rare and challenging malignancy.

## Conclusion

This case clearly demonstrates the rationale and strategy for comprehensive management of an extremely rare HP-SCNEC. The success of first-line immunotherapy combined with chemotherapy validates the potential of intensified initial treatment in controlling systemic lesions. Subsequent intracranial metastasis and successful multimodal intervention sharply highlight the limitations of current systemic therapies in conquering the CNS as the “final bastion.” This journey underscores the imperative for a dynamic perspective when treating rare, highly malignant tumors—embracing principle-driven exploratory therapies while maintaining readiness to integrate multidisciplinary approaches in response to disease evolution. The insights and lessons from this case will provide valuable reference for managing subsequent patients with similar conditions and inspire deeper research to fill gaps in this field.

## Data Availability

The original contributions presented in the study are included in the article/Supplementary Material, further inquiries can be directed to the corresponding author.
